# Long-Term Oncological Outcomes of Papillary Thyroid Cancer and Follicular Thyroid Cancer in Children: A Nationwide Population-Based Study

**DOI:** 10.3389/fendo.2022.899506

**Published:** 2022-05-04

**Authors:** Daniël J. van de Berg, Anke M. J. Kuijpers, Anton F. Engelsman, Caroline A. Drukker, Hanneke M. van Santen, Sheila C. E. J. Terwisscha van Scheltinga, A. S. Paul van Trotsenburg, Christiaan F. Mooij, Menno R. Vriens, Els J. M. Nieveen van Dijkum, Joep P. M. Derikx

**Affiliations:** ^1^ Department of Pediatric Surgery, Emma Children’s Hospital, Amsterdam University Medical Centers, University of Amsterdam, Amsterdam, Netherlands; ^2^ Department of Surgery, Amsterdam University Medical Centers, University of Amsterdam, Amsterdam, Netherlands; ^3^ Department of Surgical Oncology, Antoni van Leeuwenhoek Hospital, Amsterdam, Netherlands; ^4^ Department of Pediatric Endocrinology, Wilhelmina Children’s Hospital, Utrecht University Medical Center, University of Utrecht, Utrecht, Netherlands; ^5^ Department of Pediatric Oncology, Princess Máxima Center, Utrecht, Netherlands; ^6^ Department of Pediatric Surgical Oncology, Princess Máxima Center, Utrecht University Medical Center, University of Utrecht, Utrecht, Netherlands; ^7^ Department of Pediatric Endocrinology, Emma Children’s Hospital, Amsterdam University Medical Centers, University of Amsterdam, Amsterdam, Netherlands; ^8^ Department of Surgery, Utrecht University Medical Center, University of Utrecht, Utrecht, Netherlands

**Keywords:** papillary thyroid cancer, follicular thyroid cancer, children, long-term oncologic outcomes, pediatric

## Abstract

**Introduction:**

Pediatric thyroid carcinoma is a rare malignancy and data on long-term oncological outcomes are sparse. The aim of this study was to describe the long-term oncological outcomes of pediatric papillary thyroid carcinoma (PTC) and follicular thyroid carcinoma (FTC) in a national cohort, and to identify risk factors for recurrence.

**Methods:**

We conducted a nationwide, retrospective cohort study, in which we combined two national databases. Patients aged <18 years, diagnosed with PTC or FTC in the Netherlands between 2000 and 2016, were included. pT-stage, pN-stage, multifocality and angioinvasion were included in a Cox-regression analysis for the identification of risk factors for recurrence.

**Results:**

133 patients were included: 110 with PTC and 23 with FTC. Patients with PTC most often presented with pT2 tumors (24%) and pN1b (45%). During a median follow-up of 11.3 years, 21 patients with PTC developed a recurrence (19%). Nineteen recurrences were regional (91%) and 2 were pulmonary (9%). No risk factors for recurrence could be determined. One patient who developed pulmonary recurrence died two years later. Cause of death was not captured. Patients with FTC most often presented with pT2 tumors (57%). One patient presented with pN1b (4%). In 70%, no lymph nodes were collected. None of the patients with FTC developed a recurrence or died.

**Conclusion:**

Pediatric PTC and FTC are two distinct diseases. Recurrence in pediatric PTC is common, but in FTC it is not. Survival for both pediatric PTC and FTC is very good.

## Introduction

Pediatric thyroid cancer is rare and represents only 2.3% of all thyroid cancer diagnoses (1). In children, 85% of the cases are papillary thyroid carcinoma (PTC) and only 8% are follicular thyroid carcinoma (FTC) (2). Both types are a form of differentiated thyroid cancer (DTC) (2).

Pediatric PTC and pediatric FTC exhibit major clinical differences. Pediatric PTC is generally multifocal, bilateral and often presents with cervical lymph node metastases (3–5). Contrarily, pediatric FTC is generally unifocal and is more prone to hematogenous metastases, mainly to the lungs (3, 6). Cervical lymph node metastases are less common in children with FTC (6). In pediatric PTC, recurrence is very common, with rates ranging from 13% to 37% (4, 5, 7, 8). Conversely, long-term outcome data of pediatric FTC are very sparse. The limited data available suggest high survival rates and possibly lower to similar recurrence rates compared to pediatric PTC (6, 9).

Nevertheless, most previous studies regarding pediatric thyroid cancer included only patients with PTC or reported on patients with DTC indiscriminately, as FTC is exceedingly rare. In addition, most studies included children and adolescents up to 21 years of age.

Consequently, there is a lack of knowledge concerning the true long-term oncological outcomes of PTC and FTC, separately, in children younger than 18 years of age.

Therefore, this study aimed to describe the long-term oncological outcomes of pediatric PTC and pediatric FTC in a national cohort, and to identify risk factors for recurrence.

## Methods

This study was conducted as retrospective, nationwide, population-based cohort study and is reported according to the Strengthening the Reporting of Observational studies in Epidemiology (STROBE) guidelines (10). This study has been approved by the institutional review board of Amsterdam UMC. Nationwide data of patients with differentiated thyroid carcinoma between 0 and 18 years of age, in the period from 2000 to 2016, were collected from the ‘Netherlands Cancer Registry’ (NCR). Recurrence data were collected by matching the cohort with data from the Nationwide Network and Registry of Histo- and Cytopathology in the Netherlands (PALGA) (11). Patients who did not match the PALGA-database were excluded, because there was no confirmation of pathology or recurrence data of these patients. Using the database of PALGA and in consultation with a pathologist, all patients were retrospectively reclassified in accordance with the staging criteria of the eighth edition of the TNM classification system (12). According to the Dutch National guidelines (13, 14), all children were treated in pediatric tertiary referral centers.

Synchronous metastases were collected from the NCR-database. Cytology- or pathology proven metachronous metastases were collected from the PALGA-database. Recurrence was defined as cytology- or pathology proven recurrence after an interval of six months or longer after initial surgery. Regional recurrence was defined as cytology- or pathology proven recurrence in lymphoid- or non-lymphoid tissue in one of the cervical lymph node levels of the neck. Distant recurrence was defined as recurrence outside of the cervical lymph node levels of the neck. Last date of follow-up for survival was January 31^st^ 2021. Last date of follow-up for recurrence was March 15^th^ 2021.

Primary outcome included the disease characteristics, treatment modalities and long-term oncological outcomes of children with PTC and FTC, separately. Secondary outcome included possible risk factors for recurrence for both PTC and FTC, separately. The following variables were included in the analysis for risk factors for recurrence: pT-stage, pN-stage, multifocality and angioinvasion. Extrathyroidal extension is already imbedded as pT3b in the eight edition of the TNM classification system (12). The aforementioned variables were chosen because previous literature suggests an effect on recurrence for these variables (5, 7, 15, 16) and because we expected these to be the most relevant.

### Statistical Analysis

Patients were stratified according to tumor type. Categorical variables were expressed as numbers and percentages. Continuous variables were unanimously non-normally distributed and therefore expressed as median with interquartile range. Missing data were excluded from the analysis. A cox-regression plot was performed on recurrence data. For patients with multiple recurrences, the time to first recurrence was used. Patients without recurrence were censored at the last follow-up. Cox-regression analysis was applied on recurrence data to determine independent risk factors. Statistical significance was defined using a 2-sided α = .05 and/or 95% Confidence Interval (CI). Statistical analysis was performed using SPSS 26.0 software.

## Results

The initial cohort consisted of 135 patients. Two patients were excluded: one patient because there was no match with the PALGA-database, another patient because of uncertainty about the diagnosis. In total, 133 patients were included in this study, of which 110 children with PTC and 23 children with FTC. For patients with PTC, median age at diagnosis was 15.8 years and the male to female ratio was 1: 2.8. Two patients had a history of radiotherapy for a non-thyroid malignancy. For patients with FTC, median age at diagnosis was 16.2 years and the male to female ratio was 1: 6.7.

### Disease Characteristics

Disease characteristics are shown in **Table 1**. Most patients with PTC presented with a pT2 tumor (26 patients; 23.6%). Gross extrathyroidal extension invading strap muscle (pT3b) and gross extrathyroidal extension invading subcutaneous soft tissue, larynx, trachea, esophagus, or recurrent laryngeal nerve (pT4a) was present in 19 patients (17.3%) and 12 patients (10.9%), respectively. Multifocality of the tumor was present in 43 patients (39.1%). Angioinvasion was found in 35 patients (31.8%). Central lymph node metastases (pN1a) were present in 19 patients (17.3%) and lateral lymph node metastases (pN1b) in 49 patients (44.5%). In total, 7 patients with PTC (6.4%) presented with distant metastases.

**Table 1:** Disease characteristics of children with papillary thyroid carcinoma or follicular thyroid carcinoma.

Full cohort (N = 133 patients)		
Variables	PTC (N = 110)	FTC (N = 23)
Age, median (IQR)	15.8 (14.3 – 16.8)	16.2 (11.9 – 16.8)
Female, n (%)	81 (73.6)	20 (87.0)
History of radiotherapy, n (%)	2 (1.8)	0
		
Fine Needle Aspiration (FNA), n (%)	72 (65.5)	9 (39.1)
Bethesda classification, n (% of total FNA)		
Bethesda 1	2 (2.8)	–
Bethesda 2	2 (2.8)	–
Bethesda 3	4 (5.6)	3 (33.3)
Bethesda 4	9 (12.5)	3 (33.3)
Bethesda 5	9 (12.5)	3 (33.3)
Bethesda 6	46 (63.9)	–
		
FNA cervical lymph node, n (%)	16 (14.5)	1 (4.3)
Tumor positive, n (%)	15 (13.6)	1 (4.3)
		
No FNA of tumor or FNA of lymph node, n (%)	26 (23.6)	13 (56.5)
		
pT-stage, n (%)		
pT1a	21 (19.1)	2 (8.7)
pT1b	16 (14.5)	1 (4.3)
pT2	26 (23.6)	13 (56.5)
pT3a	13 (11.8)	5 (21.7)
pT3b	19 (17.3)	–
pT4a	12 (10.9)	2 (8.7)
pT4b	–	–
Missing values	3 (2.7)	
		
Multifocality, n (%)	43 (39.1)	1 (4.3)
Angioinvasion, n (%)	35 (31.8)	16 (69.6)
		
Tumor diameter, mm (IQR)	20 (10.3 – 39.5)	31.5 (24.8 – 44.0)
Missing values, n (%)	22 (20.0)	5 (4.6)
		
pN-stage, n (%)		
pN0	12 (10.9)	6 (26.1)
pN1a	19 (17.3)	–
pN1b	49 (44.5)	1 (4.3)
Nx	30 (27.3)	16 (69.6)
		
M1, n (%)	7 (6.4)	1 (4.3)
		
Cancer stage, n (%)		
Stage I	103 (93.6)	22 (95.7)
Stage II	7 (6.4)	1 (4.3)

Disease characteristics of children (younger than 18 years of age at diagnosis) with papillary thyroid carcinoma or follicular thyroid carcinoma.

PTC, papillary thyroid carcinoma; FTC, follicular thyroid carcinoma; n, number of patients; IQR, interquartile range; FNA, Fine Needle Aspiration.

Most patients with FTC presented with a pT2 tumor (13 patients; 56.6%). Two patients (8.7%) presented with a pT4a tumor. Multifocality of the tumor was present in one patient (4.3%). Angioinvasion was found in 16 patients (69.6%). One patient (4.3%) with FTC presented with lateral lymph node metastases. In 16 patients (69.9%), no lymph nodes were collected (Nx). Distant metastases at presentation were found in one patient (4.3%).

### Treatment Modalities

Treatment modalities are shown in **Table 2**. For patients with PTC, surgery was performed in 99.1%. Three patients (2.7%) received a hemithyroidectomy as definite surgery. All other patients received a total thyroidectomy in one or two tempi. Central- and lateral lymph node dissection was performed in 29.1%. In 9.1%, only central dissection was performed. Radioiodine remnant ablation (RRA) was given to 86.4% of the patients with PTC. Two patients solely underwent resection of a median neck cyst with an incidental finding of PTC. One patient with PTC received no treatment.

**Table 2:** Treatment modalities of children with papillary thyroid carcinoma or follicular thyroid carcinoma.

Full cohort (N = 133 patients)		
Variables	PTC (N = 110)	FTC (N = 23)
Surgery, *n (%)*	109 (99.1)	23 (100)
		
Total thyroidectomy in one tempo, *n (%)*	80 (72.7)	2 (8.7)
Total thyroidectomy in two tempi, *n (%)*	24 (21.8)	20 (87.0)
Hemithyroidectomy as only surgery, *n (%)*	3 (2.7)	1 (4.3)
Lymph node dissection, *n (%)*	42 (38.2)	1 (4.3)
Central lymph node dissection, *n (%)*	10 (9.1)	–
Central + lateral lymph node dissection, *n (%)*	32 (29.1)	1 (4.3)
		
RRA, *n (%)*	95 (86.4)	20 (87.0)
		
Other type of treatment, *n (%)*	2 (1.8)	**-**
No treatment, *n (%)*	1 (<1)	**-**

Treatment modalities of children (younger than 18 years of age at diagnosis) with papillary thyroid carcinoma or follicular thyroid carcinoma.

PTC, papillary thyroid carcinoma; FTC, follicular thyroid carcinoma; n, number of patients; RRA, radioactive Iodine remnant ablation.

All patients with FTC received surgery. One patient (4.3%) received a hemithyroidectomy as definite surgery. Twenty patients (87.0%) underwent a total thyroidectomy in two tempi. Two patients (8.7%) directly underwent a total thyroidectomy. One patient (4.3%) received central- and lateral lymph node dissection. RRA was given to 87.0% of the patients with FTC.

### Long-Term Oncological Outcomes

**Figure 1:**
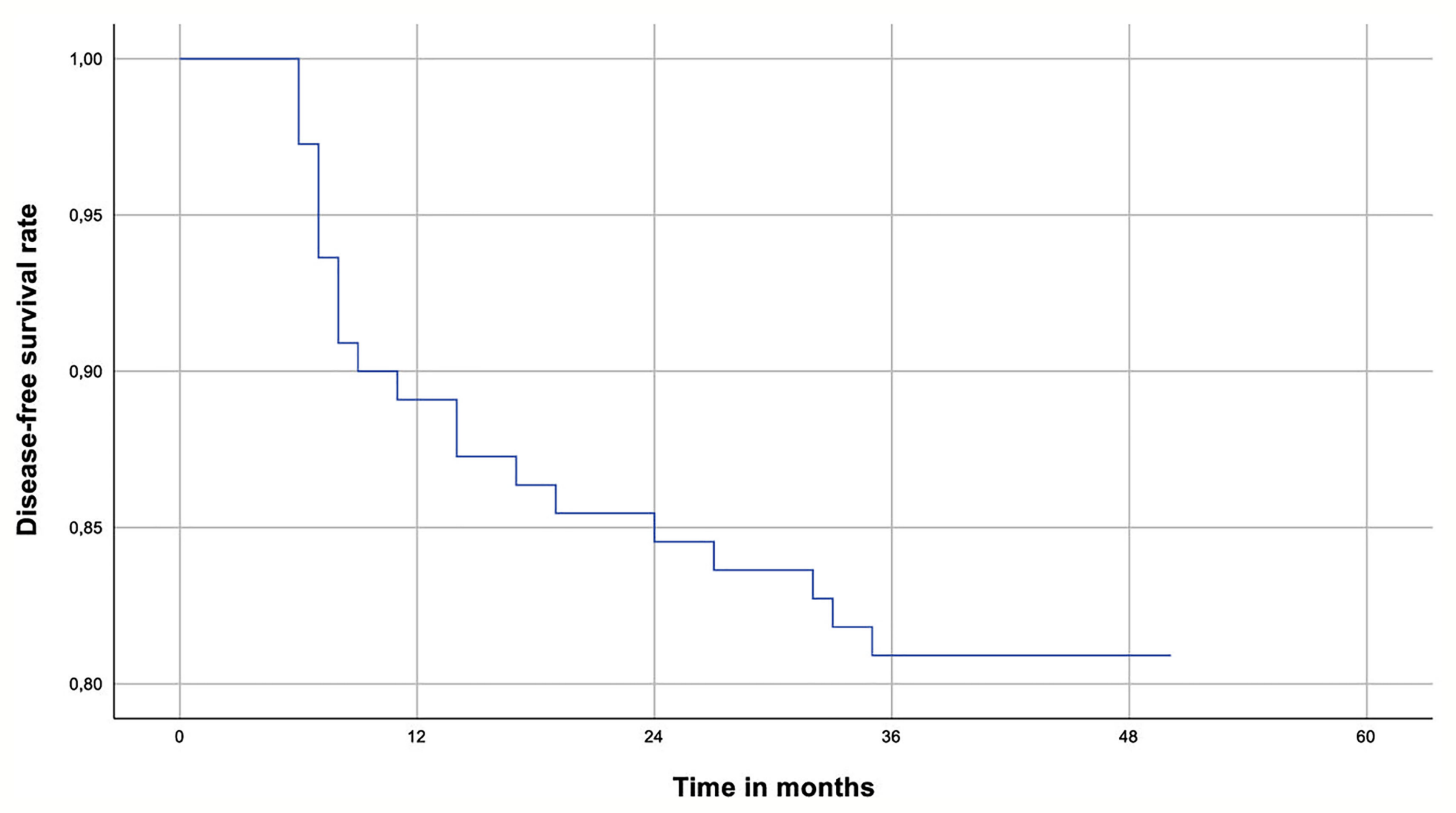
The disease-free survival rate for children with papillary thyroid carcinoma. Cox-regression plot for the disease-free survival rate in children (younger than 18 years of age at diagnosis) with papillary thyroid cancer.

Long-term oncological outcomes are shown in **Table 3**. Median follow-up for patients with PTC was 11.3 years (IQR 7.2 – 14.9). During follow-up, 21 patients (19.1%) with PTC developed a recurrence. Of these patients, nineteen developed a regional recurrence (90.5% of total recurrences). Two patients (9.5%) developed a distant recurrence (both pulmonary). Median time to recurrence was 9 months (IQR 7.0 – 21.5). All recurrences occurred within 5 years after initial surgery. During follow-up, one male patient who was diagnosed with a pT1aN0Mx PTC at age 15 years died. He was treated with a hemithyroidectomy as definite surgery and developed a pulmonary recurrence 2.5 years after initial surgery. The patient died 4 years and 9 months after initial surgery, or 2 years and 3 months after the recurrence. Because only an overall survival was captured in this database, we do not know the cause of death. The disease-free survival rate for PTC is shown in **Figure 1**.

**Table 3:** Long-term oncological outcomes of children with papillary thyroid carcinoma or follicular thyroid carcinoma.

Full cohort (N = 133 patients)		
Variables	PTC (N = 110)	FTC (N = 23)
Follow-up, median in years (IQR)	11.3 (7.2 – 14.9)	9.8 (7.3 – 12.3)
		
Recurrence, n (%)	21 (19.1)	0
5-year disease-free survival, %	80.9	100
Time to recurrence, median in months (IQR)	9 (7.0 – 21.5)	–
		
Location, n (% of total recurrences)		
Regional	19 (90.5)	–
Central lymph node	1 (4.8)	–
Lateral lymph node	14 (66.7)	–
Non-lymphoid tissue	4 (19.0)	–
Distant	2 (9.5)	–
		
Consecutive recurrences, n (%)		
1	17 (15.5)	–
2	3 (2.7)	–
3	–	–
5	1 (<1)	–
7	–	–
		
Death to all causes, n (%)	1 (<1)	0
5-year overall survival, %	99.1	100

Long-term oncological outcomes of children (younger than 18 years of age at diagnosis) with papillary thyroid carcinoma or follicular thyroid carcinoma.

PTC, papillary thyroid carcinoma; FTC, follicular thyroid carcinoma; n, number of patients; IQR, interquartile range.

Median follow-up for patients with FTC was 9.8 years (IQR 7.3 – 12.3). None of the patients with FTC developed a recurrence or died during follow-up.

### Risk Factors for Recurrence

Risk factor analysis was performed for PTC only, as no recurrences occurred in patients with FTC. Possible risk factors for recurrence in PTC are shown in **Table 4**. Cox-regression analysis for risk factors of recurrence was performed on pT-stage, pN-stage, multifocality and angioinvasion. None of the variables included were identified as risk factors.

**Table 4:** Risk factors for recurrence in children with papillary thyroid carcinoma.

Risk factors	Hazard ratio	*P* - value
pT1b	2.7 (0.4 – 16.9)	.278
pT2	1.3 (0.2 – 7.7)	.791
pT3a	2.0 (0.3 – 14.2)	.496
pT3b	2.0 (0.3 – 12.6)	.440
pT4a	5.6 (1.0 – 31.2)	.051
pN1a	1.3 (0.3 – 6.0)	.749
pN1b	2.1 (0.5 – 8.1)	.277
Multifocality	1.3 (1.0 – 1.7)	.093
Angioinvasion	0.8 (0.3 – 2.2)	.628

Cox-regression analysis for risk factors for recurrence in children (younger than 18 years at diagnosis) with papillary thyroid carcinoma.

## Discussion

In this large nationwide study, we described the disease characteristics, treatment modalities and long-term oncological outcomes of pediatric PTC and FTC in The Netherlands, and attempted to determine risk factors for recurrence. Our main findings are that recurrence in pediatric PTC is common, but in FTC it is not. Nonetheless, overall survival is very good for both PTC and FTC. During a median follow-up of 11.3 years, 19.1% of the patients with PTC developed a recurrence. Median time to recurrence was 9 months. All patients developed their recurrence within five years after initial treatment. One patient (<1%) diagnosed with a pT1aNoMx PTC died two years and three months after a pulmonary recurrence. We do not know the cause of death, as this databases did not capture cause of death. For patients with FTC, no recurrence or death occurred during a median follow-up of 9.8 years. In a Cox-regression analysis including pT-stage, pN-stage, multifocality and angioinvasion, no risk factors for recurrence of PTC could be determined.

The results of this study, in which the nationwide databases of NCR and PALGA (11) were combined, add to the sparse data on the long-term oncological outcomes of pediatric PTC and, especially, FTC in the Netherlands. In addition, the results of this study provide more insights in the disease characteristics and the treatment management of pediatric PTC and FTC.

Our finding that 62% of the patients with PTC presented with lymph node metastases, is in line with previous studies, showing rates between 58% - 86% of lymph node metastases at presentation (4, 5, 8). However, in this study, only one patient with FTC (4.3%) presented with lymph node metastases. As pediatric FTC is exceedingly rare, there are only few studies on FTC in pediatric age and most are cases series (6, 9, 17, 18). In the two most recent studies including pediatric and adolescent patients with FTC (under 21 years of age), 1 of 20 patients (5%) and none of 30 patients presented with lymph node metastases, respectively (6, 9).

During a median follow-up of 11.3 years, the recurrence rate for patients with PTC was 19%. Previous reported recurrences rates of pediatric PTC vary widely from 13% to 37%, mainly due to differences in age distribution, different definitions of recurrence and varying duration of follow-up (4, 5, 7, 8). In this study, the median time to recurrence was 9 months and all recurrences occurred within five years after initial surgery. Interestingly, though, Hay et al. (4) and Sugino et al. (7), both reported a 10-, 20- and 30-year disease-free survival of pediatric PTC and found that recurrences occurred up to 30 years after initial treatment, underlining the indolent nature of the disease. Therefore, it is possible that even with our relative long median follow-up of 11.3 years, the reported recurrence rate of 19.1% might be an underestimation of the eventual recurrence rate for pediatric PTC.

Regional recurrences in pediatric PTC seem to have no effect on disease-specific survival (4, 19), but it does necessitate additional treatment, increasing the change of postoperative complications after surgery and even secondary malignancies after RRA (20). Distant spread seems to be associated with a lower disease-free survival (4, 7), but data are controversial (19, 21).

To the best of our knowledge, only two previous studies reported long-term oncological outcomes of pediatric FTC. Enomoto et al. (6) reported outcomes of 20 children and adolescents (under 21 years old) with FTC with a median follow-up of 23.5 years and found a recurrence rate of 15% (3 of 20 children). These recurrences occurred at 6 years, 14 years and 24 years after initial surgery. The 30-year disease specific-survival was 100%. Contrarily, Spinelli et al. (9) reported outcomes of 30 children (aged younger than 19 years) with FTC with a mean follow-up of 6 years and found no recurrences and an overall survival of 100%. These results indicate that pediatric FTC, as well as pediatric PTC, is a very indolent disease. Consequently, it is possible that the follow-up of Spinelli et al. (9) and that of the present study are too short to report an accurate recurrence rate for pediatric FTC.

In the present study, we only attempted to determine risk factors for recurrence of PTC, as no recurrences occurred in patients with FTC. However, we could not determine any risk factors for recurrence of PTC (**Table 4**). This is in contrast with previous studies that found multifocality, extrathyroidal extension and lymph node metastases as risk factors for recurrence of pediatric PTC (5, 7, 15, 16). In addition, Wang et al. (16) and Rubinstein et al. (5) found younger age and non-Caucasian race as risk factors for recurrence, respectively. However, the aforementioned studies included pediatric and adolescents patients (under 21 years of age) or analyzed risk factors for DTC indiscriminately.

It is generally excepted that the clinical behavior of PTC in children is different from that of PTC in adults. Pediatric PTC tends to present with larger tumors and more often with regional or distant spread. Interestingly, 30-year cause-specific survival is better in children than in adults, despite the more advanced presentation (19). In this study, we reported high rates of regional and distant spread and high recurrence rates of pediatric PTC, which add to the belief that PTC in children might have a different clinical behavior than in adults. As FTC is very rare, studies that directly compare children with adults do not exist. However, studies of adult patients with FTC show similar patterns of metastasis compared to children, as FTC rarely metastasize to the cervical lymph nodes, but more often hematogenous to bones and lungs (22–24).

There are some limitations to this study. Firstly, Thyroglobulin levels were not captured in this database, nor were Iodine scans. Therefore, it is possible that we missed some patients that presented with biochemical recurrences or distant recurrences, which are not always confirmed with histopathology. In that case, the recurrence rate of 19.1% would be an underestimation. In addition, it is possible that some patients with an early recurrence presented with persistent disease rather than recurrent disease. We could not differentiate between an early recurrence or persistent disease, as we had no Tg-values or Iodine scans to define a period of no evidence of disease. However, it is expected that any residual disease is treated within six months of diagnosis. Secondly, cause of death was not captured in this database. Therefore, we cannot make a definite statement about the cause of death of the patient that died two years and three months after a pulmonary recurrence of PTC. Thirdly, our long follow-up of 11.3 years for PTC and 9.8 years for FTC, respectively, might not be sufficient to determine all recurrences and mortality, as pediatric PTC and FTC are very indolent diseases. However, it is expected that only a very small proportion of the patients develops a recurrence or dies after 10 years of follow-up, as all recurrences and mortality in this study occurred during the first 5 years after initial surgery.

## Conclusion

In conclusion, the results of this nationwide study show that pediatric PTC and FTC are two very distinct diseases with different long-term oncological outcomes. Recurrence is very common in children with PTC. Contrarily, during follow-up, no recurrences occurred in children with FTC. Both pediatric PTC and FTC have a very high survival rate. Only one patient with PTC died to any cause two years and three months after a pulmonary recurrence. In this study, no risk factors for recurrence could be determined. Based on our results, we propose that pediatric PTC and FTC should not be grouped together as DTC indiscriminately when determining long-term oncological outcomes. More research with a follow-up longer than 10 years should be conducted to reveal the oncological outcomes of pediatric FTC. As the survival rate in pediatric PTC is very high, future research should focus on the factors differentiating between patients at risk for recurrence and patients not at risk. Conducting these studies could lead to more a personalized treatment of both pediatric PTC and FTC, thereby improving the oncological outcomes of our high risk patients while reducing overtreatment of our low risk patients.

## Authors Contributions

CD and JD collected the data from IKNL for this study. DB, AK, CD, JD collected the data from PALGA for this study. DB performed the analyses under supervision of AK and JD. DB took the lead in writing the manuscript. AE, CD, HS, ST, AT, CM, MV and EN all critically reviewed and improved the manuscript. All authors agreed with the publication of the manuscript in its current form.

## Data Availability Statement

The original contributions presented in the study are included in the article/supplementary material. Further inquiries can be directed to the corresponding author.

## Ethics Statement

The studies involving human participants were reviewed and approved by Medisch Ethische Toetsings Commissie AMC. Written informed consent from the participants’ legal guardian/next of kin was not required to participate in this study in accordance with the national legislation and the institutional requirements.

## Funding

JD and AT received a ‘Steun Stichting Emma Kinderziekenhuis’ grant for research in the field of differentiated thyroid carcinoma in children, adolescents and young adults. Grant number: WAR2020-10.

## Conflict of Interest

The authors declare that the research was conducted in the absence of any commercial or financial relationships that could be construed as a potential conflict of interest.

## Publisher’s Note

All claims expressed in this article are solely those of the authors and do not necessarily represent those of their affiliated organizations, or those of the publisher, the editors and the reviewers. Any product that may be evaluated in this article, or claim that may be made by its manufacturer, is not guaranteed or endorsed by the publisher.
